# Aspirin effect on the incidence of major adverse cardiovascular events in patients with diabetes mellitus: a systematic review and meta-analysis

**DOI:** 10.1186/1475-2840-10-25

**Published:** 2011-04-01

**Authors:** Sonia Butalia, Alexander A Leung, William A Ghali, Doreen M Rabi

**Affiliations:** 1 Department of Medicine, Faculty of Medicine, University of Calgary, Calgary, Alberta, Canada; 2 Department of Community Health Sciences, Faculty of Medicine, University of Calgary, Calgary, Alberta, Canada; 3 Department of Cardiac Sciences, Faculty of Medicine, University of Calgary, Calgary, Alberta, Canada

## Abstract

**Background:**

Aspirin has been recommended for the prevention of major adverse cardiovascular events (MACE, composite of non-fatal myocardial infarction, non-fatal stroke, and cardiovascular death) in diabetic patients without previous cardiovascular disease. However, recent meta-analyses have prompted re-evaluation of this practice. The study objective was to evaluate the relative and absolute benefits and harms of aspirin for the prevention of incident MACE in patients with diabetes.

**Methods:**

We performed a systematic review and meta-analysis on seven studies (N = 11,618) reporting on the use of aspirin for the primary prevention of MACE in patients with diabetes. Two reviewers conducted a systematic search of electronic databases (MEDLINE, EMBASE, the Cochrane Library, and BIOSIS) and hand searched bibliographies and clinical trial registries. Reviewers extracted data in duplicate, evaluated the quality of the trials, and calculated pooled estimates.

**Results:**

A total of 11,618 participants were included in the analysis. The overall risk ratio (RR) for MACE was 0.91 (95% confidence intervals, CI, 0.82-1.00) with little heterogeneity among trials (I^2 ^0.0%). Secondary outcomes of interest included myocardial infarction (RR, 0.85; 95% CI, 0.66-1.10), stroke (RR, 0.84; 95% CI, 0.64-1.11), cardiovascular death (RR, 0.95; 95% CI, 0.71-1.27), and all-cause mortality (RR, 0.95; 95% CI, 0.85-1.06). There were higher rates of hemorrhagic and gastrointestinal events. In absolute terms, these relative risks indicate that for every 10,000 diabetic patients treated with aspirin, 109 MACE may be prevented at the expense of 19 major bleeding events (with the caveat that the relative risk for the latter is not statistically significant).

**Conclusions:**

The studies reviewed suggest that aspirin reduces the risk of MACE in patients with diabetes without cardiovascular disease, while also causing a trend toward higher rates of bleeding and gastrointestinal complications. These findings and our absolute benefit and risk calculations suggest that those with diabetes but without cardiovascular disease lie somewhere between primary and secondary prevention patients on the spectrum of benefit and risk. This underscores the importance of considering individual risk in clinical decision making regarding aspirin in those with diabetes.

## Background

Cardiovascular disease is the leading cause of mortality in patients with diabetes, accounting for an estimated 65-80% of deaths in these patients [1-3]. Although there have been substantial reductions in cardiovascular-related morbidity and mortality in the general population over the last 40 years attributed to improved treatment of cardiovascular risk factors and disease, the same magnitude of benefit has not been observed in those with diabetes mellitus [4,5]. It remains unclear why certain interventions that benefit the general population may be less effective for patients with diabetes.

A landmark observational study suggested that patients with diabetes without prior myocardial infarction had a similar risk of coronary heart disease as patients with prior myocardial infarction without diabetes [6]. As a result of the ideas introduced by this study, significant interest emerged for the widespread implementation of interventions for lowering cardiovascular risk in patients with diabetes, such as the use of aspirin therapy. However, as others have pointed out, the widespread use of aspirin in patients with diabetes mainly reflect "extrapolations from other high risk groups... rather than on a comprehensive review of pertinent data" [7].

Several meta-analyses have explored the benefit of aspirin therapy in the primary prevention of major adverse cardiovascular events (MACE) among patients with diabetes [8-13], and concluded that there is insufficient evidence to routinely recommend aspirin therapy in patients with diabetes without known cardiovascular disease [8-10]. While these reviews provided a robust statistical summary of relative risk, some [8-10] did not report or assess aspirin effects on absolute event rates for cardiovascular and bleeding events. The latter is crucial to considering the trade-off of benefit and harm associated with aspirin. Moreover, current estimates of benefit still remain uncertain. A collaborative meta-analysis of individual patient data suggested that there was a net benefit of aspirin therapy for diabetic patients in preventing serious vascular events in the six primary prevention trials reviewed. Yet, it was noted that the results from three other primary prevention trials in diabetes did not demonstrate the same benefit [13].

In light of this uncertainty, we conducted a systematic review and meta-analysis that included data from primary prevention trials that enrolled patients from the general population as well as trials selecting for diabetic patients, as a few of these [14-16] were not previously incorporated in some of the earlier reviews [8,10], and we sought to additionally quantify treatment effects in absolute terms to better-inform readers of the risk-benefit trade-off of aspirin therapy in patients with diabetes.

## Methods

### Search strategy

A comprehensive search was performed in MEDLINE (1950 to February 2011), PubMed, EMBASE (1980 to February 2011), and the Cochrane Library (including the Cochrane Database of Systematic Reviews, Database of Abstracts of Reviews of Effects, and Cochrane Central Register of Controlled Trials), and BIOSIS. Language restrictions were not applied, but our search was limited to human studies and randomized clinical trials using the Cochrane Collaboration filter [17]. We searched titles and abstracts with the terms "diabetes mellitus," "primary prevention," and "aspirin" as keywords (*exp*), and Medical Subject Headings (MeSH). The Boolean term "AND" was used to combine "diabetes mellitus" and "aspirin" as well as to combine "primary prevention" and "aspirin." The final results were then combined with the Boolean operator "OR." Bibliographies of identified studies and recent related meta-analyses were hand-searched. Clinical trial registries (clinicaltrials.gov, isrctn.com, and controlled-trials.com) were also searched for ongoing and unpublished studies. Details of our search strategy are included in Additional file 1.

### Study selection and data abstraction

SB and AAL independently reviewed the retrieved titles and abstracts and selected all studies reporting on the use of aspirin, the primary prevention of cardiovascular disease, and with original data reporting on a variety of cardiovascular events (see below). Full text review was then independently performed by SB and AAL for inclusion of randomized trials that compared aspirin therapy versus a cardiac-neutral comparator (e.g. placebo, vitamins), which enrolled adults (≥18 years old), and patients with diabetes mellitus without previous historical or clinical evidence of cardiovascular disease. Studies were excluded if they were secondary publications of trials already included in the analysis, if the trial duration was twelve-months or less, or if the data (as published) could not be extracted for diabetes-specific outcomes. Disagreements in study inclusion between reviewers were resolved by consensus, and quantified.

All outcome data were extracted by SB and AAL independently, with subsequent discussion of any discrepancies. Data were collected on baseline patient characteristics, aspirin dosing and frequency, and various cardiovascular event rates (cardiovascular death, myocardial infarction, stroke, all-cause mortality, hemorrhage, gastrointestinal bleeding, and other gastrointestinal events not resulting in bleeding). Outcomes from each study were extracted in intention-to-treat categories, rather than per-protocol categories. Quality assessment was performed by extracting information on key study validity criteria [18] and a Jadad score was calculated for each study [19].

### Statistical analysis

We assessed and quantified statistical heterogeneity for each outcome of interest using Cochran's Q test and the I^2 ^statistic, respectively [20]. Analyses for the primary outcome of interest, MACE (defined as a composite of non-fatal myocardial infarction, non-fatal ischemic stroke, and cardiovascular death resulting from myocardial infarction and ischemic stroke), and all-cause mortality were performed using the Mantel-Haenszel fixed effects model. The remainder of our analyses were performed using the DerSimonian and Laird random effects model because some heterogeneity was present. We calculated risk ratios (RR) and 95% confidence intervals (95% CIs) for MACE, total myocardial infarction, total stroke, cardiovascular death, all-cause mortality, hemorrhage, gastrointestinal bleeding, and other gastrointestinal events not resulting in bleeding. We subsequently contextualized our results by calculating the absolute risk reduction (ARR, derived by calculating the difference between the combined control arm event rate and the product of the pooled relative risk and the combined control arm event rate), the number need to treat (NNT) and then compared our estimated to published data. The balance of risk and benefit was further represented by the "likelihood of being helped versus harmed" metric (LHH, a ratio of number needed to harm, as estimated by the Antithrombotic Trialists' (ATT) Collaboration [13], divided by the NNT) [21,22].

Subgroup analyses were conducted to examine the effects of aspirin dosage, allocation concealment, randomization, blinding, and whether a trial exclusively enrolled patients with diabetes on the pooled RR. We performed meta-regression analyses using maximum likelihood estimation. Assessment for publication bias was performed for the main outcome of interest with Egger's linear regression test [23,24]. Analyses were performed using Stata version 11 (StataCorp, College Station, Texas). The study is reported according to PRISMA guidelines [18].

## Results

### Study selection and evaluation

**Figure 1:**
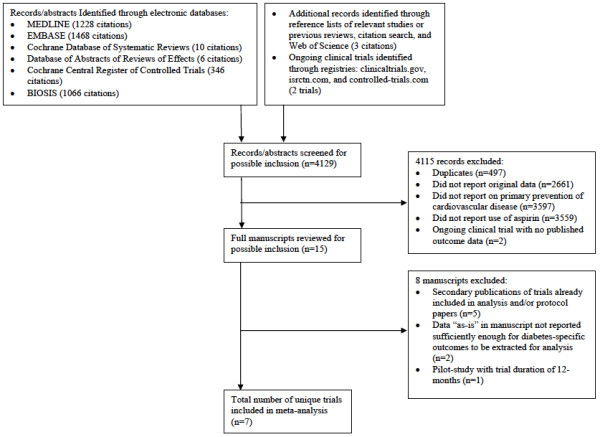
**Study flow diagram**.

Of the 4129 citations identified in our search, fifteen were identified for full-text review, and a total of seven unique trials were eligible for inclusion in this study (see Figure 1 for PRISMA flow chart). Follow-up searches failed to identify any additional trials that met inclusion criteria. Disagreement among the two reviewers regarding eligibility of studies occurred on only five occasions (κ score = 0.89).

### Studies included in the systematic review

Summary data from the seven randomized clinical trials (n = 11,618) are presented in Table 1[14-16,25-29]. The Physicians' Health Study (PHS) recruited male physicians, approximately 2% of whom had diabetes [29], and the Women's Health Study (WHS) enrolled women with approximately 3% of trial participants identified to have diabetes [27]. The Hypertension Optimal Treatment (HOT) trial studied whether the addition of aspirin to antihypertensive treatment reduced the risk of MACE compared with placebo [14,15]. The remaining four trials [16,25,26,28] exclusively studied patients with diabetes, with two trials studying type 2 diabetes only [25,28], and two trials incorporating patients with type 1 or type 2 diabetes [16,26]. Approximately half of the participants in the Early Treatment Diabetic Retinopathy Study (ETDRS) were reported to have a history of cardiovascular disease (i.e. self-reported history of coronary disease, heart failure, or peripheral arterial disease, as well as patients on any beta-adrenergic antagonist, anti-hypertensive agent, antiarrhythmic agents, digitalis, or nitrates) [16]. The remaining six trials (as reported) did not enroll any patients with overt cardiovascular disease. There was substantial variability in aspirin dosages with higher doses of 325 mg every other day in the PHS [29] and 650 mg daily in the ETDRS [16]. Aspirin doses of 100 mg or less per day were used in the remaining five trials: Primary Prevention Project (PPP) [28], WHS [27], HOT [14,15], Prevention and Progression of Arterial Disease and Diabetes Trial (POPADAD) [26], and Japanese Primary Prevention of Atherosclerosis with Aspirin for Diabetes (JPAD) [25]. Trial duration ranged from 3.6 years [28] to 10.1 years [27].

**Table 1:** Design and description of trials of aspirin therapy included in systematic review and meta-analysis

Study and Year	Study design	Patient Population	ASA Dose	Duration of ASA therapy (years)	Exclusively enrolled patients with DM	# of people with DM (% of sample)	Duration of DM	Mean HbA1C %	Primary outcome measures
**PHS 1989**	Randomized, double blind*, placebo controlled trial	Male physicians	325 mg every other day	5	No	533 (2.4)	NR	NR	CV mortality

**ETDRS 1992**	Randomized, double blind*, placebo controlled trial	Men and women with type 1 or type 2 DM	650 mg every day	5	Yes	3711 (100)	Greater than 80% for >10 years	>40% with HbA1C >10%	All cause mortality

**HOT 1998**	Randomized, double blind*, placebo controlled trial##	Men and women with hypertension	75 mg every day	3.8	No	1501 (8)	NR	NR	Composite endpoint of CV death, MI, or stroke

**PPP 2003**	Randomized, open label trial with 2 × 2 factorial design	Men and women with DM age >50 with ≥ 1 RF for CVD	100 mg every day	3.6	Yes	1031 (100)	NR	7.6, 7.6	Composite end point CV death, MI, or stroke

**WHS 2005**	Randomized, double blind*, 2 × 2 factorial, placebo controlled trial	Women	100 mg every other day	10.1	No	1027 (2.6)	NR	NR	Composite end point of non-fatal MI, non-fatal stroke, death from CVD

**POPADAD2008**	Randomized, double blind*, 2 × 2 factorial, placebo controlled trial	Men and Women with type 1 or 2 DM and an ABI ≤ 0.99 but no symptomatic CVD	100 mg every day	6.7 **	Yes	1276 (100)	Greater than 6 years in each group	7.9-8.0	Death from CHD or stroke, non-fatal MI or stroke, or amputation above ankle for critical limb ischemia and death from CHD

**JPAD 2008**	Randomized, open label controlled trial	Men and women with type 2 DM without history of atherosclerotic disease	81 or 100 mg every day	4.4	Yes	2539 (100)	7.3, 6.7***	7.1, 7.0	Atherosclerotic events including fatal or non-fatal heart disease, fatal or non-fatal stroke, and PAD

PHS: Physicians' Health Study, ETDRS: Early Treatment Diabetic Retinopathy Study, HOT: Hypertension Optimal Treatment, PPP: Primary Prevention Project, WHS: Women's' Health Study, POPADAD: Prevention and Progression of Arterial Disease and Diabetes Trial, JPAD: Japanese Primary Prevention of Atherosclerosis with Aspirin for Diabetes, DM: diabetes, ASA: aspirin, RF: risk factor, CVD: cardiovascular disease, ABI: ankle brachial index, NR: data not reported, * patients and outcomes assessors, ## Patients were also randomly assigned to one of three diastolic blood pressure target groups, ** median, ***: ASA, control group median years, CV: cardiovascular, MI: myocardial infarction, CHD: coronary heart disease, PAD: peripheral arterial disease

A summary of study quality indicators is presented in Table 2. Randomization occurred in all seven studies, but the use of allocation concealment was clearly stated in only five of the trials [14-16,26,28,29]. All seven studies had a Jadad score of three or greater. Intention-to-treat analysis was explicit in four trials [16,25,27,28] and loss to follow-up was accounted for in all trials except one [28]. Treatment in three studies was randomly assigned according to a 2 × 2 factorial design [26-28]. The WHS [27], PPP [28], and POPADAD [26] trials also had a vitamin or anti-oxidant component. Two trials were open-labeled [25,28]. None of the trials appeared to have substantial baseline differences between patients allocated to aspirin therapy versus the comparator-arm.

**Table 2:** Summary of quality indicators for studies assessing aspirin in patients with diabetes for the primary prevention of major adverse cardiovascular events

Study, Year	Allocation Concealment	Blinding of participants and outcome-assessors	Placebo - controlled	Intention to Treat Analysis	Lost to Follow up Accounted	Potential Baseline Difference	JADAD Score (Range 0-5)
PHS, 1989#	Yes	Yes	Yes	Undetermined	Yes	No	5

ETDRS, 1992	Yes	Yes	Yes	Yes	Yes	No	4

HOT, 1998	Yes	Yes	Yes	Undetermined	Yes	No	4

PPP, 2003#	Yes	No	No*	Yes	Undetermined	No	3

WHS, 2005#	Undetermined	Yes	Yes	Yes	Yes	No	4

POPADAD, 2008	Yes	Yes	Yes**	Undetermined	Yes	No	5

JPAD, 2008	No	No	No	Yes	Yes	No	3

PHS: Physicians' Health Study, ETDRS: Early Treatment Diabetic Retinopathy Study, HOT: Hypertension Optimal Treatment, PPP: Primary Prevention Project, WHS: Women's' Health Study, POPADAD: Prevention and Progression of Arterial Disease and Diabetes Trial, JPAD: Japanese Primary Prevention of Atherosclerosis with Aspirin for Diabetes, # methods papers reviewed, * and/or comparator of vitamin E 300 mg once daily, **and/or comparator of anti-oxidant tablet

### Major adverse cardiovascular events

**Figure 2:**
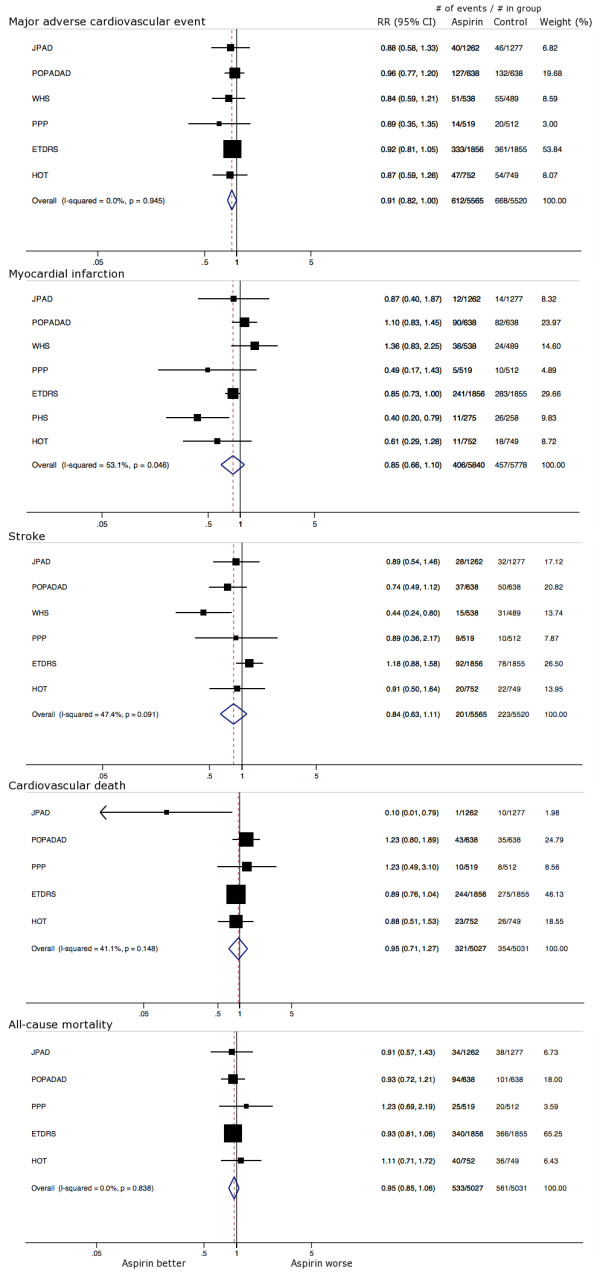
**Effect of aspirin on the primary prevention of myocardial infarction (non-fatal and fatal), stroke (non-fatal and fatal), cardiovascular death, major adverse cardiovascular events (a composite of non-fatal myocardial infarction, non-fatal stroke, and cardiovascular death), and all-cause mortality in patients with diabetes**. Relative risks (RRs) are indicated by squares with the relative weight of each trial represented by the size of each square. 95% confidence intervals (CIs) are indicated by horizontal lines. Pooled risk estimates and corresponding 95% CIs are represented by diamonds. Squares or diamonds to the left of the solid line indicate benefit with aspirin therapy. JPAD = Japanese Primary Prevention of Atherosclerosis with Aspirin for Diabetes; POPADAD = Prevention Of Progression of Arterial Disease And Diabetes; WHS = Women's Health Study; PPP = Primary Prevention Project; ETDRS = Early Treatment Diabetic Retinopathy Study; HOT = Hypertension Optimal Treatment; and PHS = Physicians' Health Study.

Six trials reported data on MACE [14-16,25-28]. A total of 612 MACE occurred among the 5565 participants with diabetes treated with aspirin compared to 668 MACE among 5520 diabetic participants in the control group (Table 3; Figure 2). Tests for statistical heterogeneity of results across the six trials revealed homogeneity (I^2 ^= 0.0%; p = 0.945). None of the individual trials reported a significantly decreased risk of MACE among participants assigned to aspirin. However, the pooled effect estimate was very nearly significant with a relative risk (RR) of 0.91 (95% CI, 0.82 to 1.00).

**Table 3:** Cardiovascular and mortality outcomes in studies assessing aspirin in patients with diabetes for the primary prevention of major adverse cardiovascular events

Study and Year	Total Patients with Diabetes	MACE (ASA)	MACE (Control)	Total Mortality (ASA)	Total Mortality (Control)	CV Mortality (ASA)	CV Mortality (Control)	Total MI* (ASA)	Total MI* (Control)	Total Stroke* (ASA)	Total Stroke* (Control)
PHS, 1989	533	-	-	-	-	-	-	11	26	-	-

ETDRS, 1992	3711	333	361	340	366	244	275	241	283	92	78

HOT, 1998	1501	47	54	40	36	23	26	11	18	20	22

PPP, 2003	1031	14	20	25	20	10	8	5	10	9	10

WHS, 2005	1027	51	55	-	-	-	-	36	24	15	31

POPADAD, 2008	1276	127	132	94	101	43	35	90	82	37	50

JPAD, 2008	2539	40	46	34	38	1	10	12	14	28	32

MACE: major adverse cardiovascular events, ASA: aspirin, CV: cardiovascular, MI: myocardial infarction, *: non-fatal and fatal, " - ": not reported, PHS: Physicians' Health Study, ETDRS: Early Treatment Diabetic Retinopathy Study, HOT: Hypertension Optimal Treatment, PPP: Primary Prevention Project, WHS: Women's' Health Study, POPADAD: Prevention and Progression of Arterial Disease and Diabetes Trial, JPAD: Japanese Primary Prevention of Atherosclerosis with Aspirin for Diabetes.

### Myocardial infarction

A total of 406 myocardial infarctions (non-fatal and fatal) was documented among 5840 participants in the aspirin group compared to 457 myocardial infarctions among the 5778 participants in the control group across the seven trials (Table 3; Figure 2) [14-16,25-29]. Heterogeneity of the trial results was moderate to high (I^2 ^= 53.1%; p = 0.046), which may be in part due to the WHS [27] (15% weight) and the PHS [29] (10% weight) trials, which enrolled exclusively women and men, respectively. The pooled risk estimate of the total available data was non-significant (RR, 0.85; 95% CI, 0.66 to 1.10). After exclusion of WHS [27] and PHS [29], heterogeneity substantially decreased between trials (I^2 ^= 14.2%; p = 0.324) with no substantial change in the overall effect estimate (RR, 0.89; 95% CI, 0.75 to 1.06).

### Ischemic stroke

Among the 5565 participants assigned to aspirin therapy, a total (non-fatal ischemic and fatal ischemic) of 201 strokes occurred, compared to 223 strokes in the control group of 5520 participants (Table 3; Figure 2) [11-13,20-23]. Moderate amounts of heterogeneity between study results were quantified (I^2 ^= 47.4%; p = 0.091). Some heterogeneity may be explained by the WHS (14% weight), a trial of women only and with the greatest relative risk reduction of strokes with aspirin [27]. The pooled RR of the results from the five trials was non-significant (0.84; 95% CI, 0.63 to 1.11). After exclusion of the WHS [27], heterogeneity diminished (I^2 ^= 0.0%; p = 0.468) and the pooled effect estimate remained non-significant.

### Cardiovascular death

Death directly attributed to cardiovascular disease was found in 321 participants treated with aspirin, and 354 in the control group across five trials (Table 3; Figure 2) [14-16,25,26,28]. Only JPAD, a trial of Japanese patients, reported a statistically significant risk reduction with aspirin therapy [25]. Moderate amounts of heterogeneity were quantified in this analysis (I^2 ^= 41.1%; p = 0.148). The pooled estimate of RR of the results from the four trials was non-significant (0.95; 95% CI, 0.71 to 1.27). Although further exclusion of JPAD [25] resulted in a reduction in statistical heterogeneity (I^2 ^= 0.0%, p = 0.507), the overall effect estimate remained broadly similar (RR, 0.93; 95% CI, 0.80 to 1.07).

### All-cause mortality

A total of 533 deaths were recorded among 5027 participants treated with aspirin compared to 561 deaths in the control arm with 5031 participants (Table 3; Figure 2) [14-16,25,26,28]. None of the trials reported significant reductions in risk of death associated with aspirin therapy. Heterogeneity was non-significant between trials (I^2 ^= 0.0%; p = 0.838). The pooled effect estimate was also non-significant (RR, 0.95; 95% CI, 0.85 to 1.06).

### Drug-related side effects

Hemorrhagic complications were uncommon. A total of 81 bleeding events occurred among 3637 participants treated with aspirin compared to 48 hemorrhagic complications among the 3644 participants treated with placebo, for a pooled RR risk of 2.50 (95% CI, 0.77-8.10) (Table 4). 48 cases of gastrointestinal bleeding were documented among 2419 participants on aspirin compared to 36 cases among 2427 in the control group (RR 2.13, 95% CI 0.63-7.25). Gastrointestinal events not resulting in bleeding (such as dyspepsia, non-bleeding peptic ulcers, or gastritis) occurred in 120 of 1900 participants on aspirin compared to 98 of 1915 participants in the control arm (RR 2.92 (0.17-50.23). None of these differences were statistically significant (Table 4), but statistical power was limited given the relatively low frequency of these adverse events.

**Table 4:** Risk of adverse events in studies assessing aspirin in patients with diabetes for the primary prevention of major adverse cardiovascular events

Study and Year	Total Patients with Diabetes	All Bleeding (ASA)	All Bleeding (Control)	All GI Bleeding (ASA)	All GI Bleeding (Control)	Non-bleeding GI symptoms (ASA)	Non-bleeding GI symptoms (Control)
PHS, 1989	533	-	-	-	-	-	-

ETDRS, 1992	3711	37	37	-	-	-	-

HOT, 1998	1501	-	-	-	-	-	-

PPP, 2003	1031	10	1	8	1	-	-

WHS, 2005	1027	-	-	-	-	-	-

POPADAD, 2008	1276	-	-	28	31	73	94

JPAD, 2008	2539	34	10	12	4	47	4

Number of Events/Number of Participants	-	81/3637	48/3644	48/2419	36/2427	120/1900	98/1915

Pooled RR (95% CI)	-	2.50 (0.77-8.10)		2.13 (0.63-7.25)		2.92 (0.17-50.23)	

ASA: aspirin, GI: gastrointestinal, PHS: Physicians' Health Study, ETDRS: Early Treatment Diabetic Retinopathy Study, HOT: Hypertension Optimal Treatment, PPP: Primary Prevention Project, WHS: Women's' Health Study, POPADAD: Prevention and Progression of Arterial Disease and Diabetes Trial, JPAD: Japanese Primary Prevention of Atherosclerosis with Aspirin for Diabetes, RR = relative risk (RRs >1 indicate increase risk of an adverse event associated with aspirin).

### Absolute benefit and harm

The ARR of MACE in the reviewed studies is 1.09% associated with aspirin therapy with a corresponding number needed to treat (NNT) of 92 to prevent one major cardiovascular event. The estimate of the LHH is 6 (range of 2-8).

### Subgroup analysis

Subgroup analysis was performed to explore potential sources of variability between trials. There was no statistical significant effect on the pooled RR when analyzed according to aspirin dosage, allocation concealment, or whether a trial exclusively enrolled patients with diabetes. Randomization and blinding (of participants or outcome assessors) were also found to be non-significant covariates of heterogeneity (data not shown).

### Publication bias

We assessed for publication bias by Egger's linear regression test. The β-coefficient of the bias estimate was not statistically significant (β-coefficient = -0.72; 95% CI, -1.52 to 0.09; p = 0.07). The PHS trial was not included in the test for publication bias because MACE data were not reported [29].

## Discussion

This meta-analysis of seven randomized clinical trials, combining data from 11,618 participants, indicates that aspirin therapy in patients with diabetes leads to a 9% relative reduction in the risk of major adverse cardiovascular events, a pooled risk reduction estimate that verges on statistical significance. The point estimates of aspirin effect on each of the components of the MACE composite endpoint also all favor aspirin therapy, suggesting a signal of benefit, though the analyses for each of the individual endpoints does not meet statistical significance. Drug-related side effects were relatively uncommon and not consistently reported across trials, but point estimates of effect suggest a trend toward increased risk of hemorrhage and adverse gastrointestinal events among participants treated with aspirin.

These results are not inherently surprising and while consistent with other recently published meta-analyses addressing the same clinical question [8-13], also extend findings of prior reviews. It is not surprising that several investigators have concurrently conducted systematic reviews to help answer such an important, yet unresolved question as "Is aspirin effective at preventing cardiovascular events in patients with diabetes who have not had a prior event?" While other investigators reviewing this topic have chosen to interpret statistically non-significant benefit as no evidence of benefit [8,9], we view the results differently and extend the findings to provide (below) estimates of the balance of *absolute *risks and benefits.

It has been clearly demonstrated in a variety of patient populations that aspirin significantly decreases the risk of MACE while increasing the risk of bleeding complications [13]. Aspirin use has established benefits and associated harms and it is the relative trade-off of cardiovascular events prevented versus bleeding events caused by aspirin that needs to be carefully considered on the basis of patient characteristics and individualized estimates of cardiovascular risk [13]. Patients with established cardiovascular disease are at greatest risk for MACE and death, and thus benefit the most from aspirin therapy. In contrast, the absolute benefit of aspirin is substantially less pronounced in patients estimated to be at lower risk for the primary prevention of cardiovascular disease [13,30,31].

The pressing question is thus to consider where on the spectrum of absolute benefit and harm patients with diabetes without known existing cardiovascular disease fall? To date, the answer has been somewhat elusive, though our results shed some light on this question. The ARR of MACE in the studies that we reviewed is 1.09% associated with aspirin therapy with a corresponding NNT of 92 to prevent one major cardiovascular event. In comparison, patients with diabetes at greatest risk for MACE (i.e. risk approaching those in the general population with established cardiovascular disease) may have an associated NNT of 67; conversely, the NNT may be as high as 1667 in patients at lowest risk (i.e. risk identical to those in the general population without cardiovascular disease) [13]. Estimates of number needed to harm (NNH) for major bleeding range from 526 to 3333 in patients treated with aspirin for secondary and primary prevention, respectively [13]. The balance of risk and benefit, represented by the "likelihood of being helped versus harmed" metric (LHH), was estimated to be 6 (range of 2-8) with a more favorable risk-benefit balance with greater baseline risk. As such, for every 10,000 diabetic patients treated with aspirin, approximately 109 MACE may be prevented at the expense of 19 major bleeding events with no significant overall mortality benefit.

In this context, the fundamental question relating to patients with diabetes without cardiovascular disease is consideration of whether they are more like the general population without cardiovascular disease (i.e. "primary prevention cases"), or more in keeping (in terms of risk) with patients who have been included in secondary prevention studies. Our calculation of absolute benefit would suggest that those with diabetes but without prior cardiovascular disease lie in an intermediate position between a primary and secondary prevention on the spectrum of risk and benefit.

This invokes the controversial question of whether diabetes is a "coronary disease equivalent." There is widespread acceptance that diabetes is a major risk factor for cardiovascular disease [2,32-34], but there have been varying estimates of absolute risk [3]. In 1998, Haffner *et al. *found that patients with diabetes without prior myocardial infarction had a similar risk of coronary heart disease as patients with prior myocardial infarction without diabetes (i.e. affirming the "coronary disease equivalent" theory) [6]. However, at least twelve subsequent observational studies have reported conflicting results compared to the initial Finnish study [3], and a recent systematic review has presented data arguing against the notion of diabetes being a coronary-risk equivalent [3]. Although the literature uniformly agrees that patients with diabetes are at increased risk of cardiovascular events, it now suggests that the absolute risk is less than previously thought, and may explain why the benefit of some evidence-based "secondary prevention" treatments like aspirin are less marked when applied to patients with diabetes in a population-based manner.

In considering our review findings, there is a need to consider pathophysiological factors and a potential modifying effect of diabetes on aspirin efficacy. Patients with diabetes are felt to be at higher risk of atherothrombotic events because of endothelial dysfunction, impaired fibrinolysis, increased levels of circulating coagulation factors, and high platelet reactivity [35]. However, aspirin may have a dampened effect in some diabetic patients in preventing platelet aggregation [36-38]. Emerging experimental studies are now demonstrating the phenomenon of "resistance" to aspirin, which appears to have a greater prevalence among patients with diabetes [35]. Aspirin resistance has been correlated to long-term adverse outcomes with increased coronary heart disease, stroke, and peripheral vascular disease risk [35]. Whether higher doses of aspirin may translate to improved clinical outcomes in the setting of aspirin resistance, or if another antiplatelet agent is superior remains unclear.

There are currently two major ongoing trials, collectively enrolling more than 15,000 participants, evaluating the role of aspirin in patients with diabetes without prior cardiovascular events: Aspirin and Simvastatin Combination for Cardiovascular Events Prevention Trial in Diabetes (ACCEPT-D) [39] and A Study of Cardiovascular Events in Diabetes (ASCEND) [40]. In addition to exploring the role of aspirin in the prevention of cardiovascular events in patients with diabetes, these trials will help elucidate which patients with may be at greatest risk, and thus derive the most benefit from aspirin therapy.

### Strengths and limitations

There are limitations to our study. Firstly, we were only able to incorporate a total of seven trials in our analysis, and quantified moderate amounts of heterogeneity among trials for the outcomes of interest. The trials of interest varied greatly when comparing patient populations, geographic locale, years of study, and design. Moreover, pooling of data was limited by inconsistent reporting of baseline characteristics and outcomes, differences in the metrics of reported values, and variability in definitions of the measured outcomes of interest (e.g. major bleeding). Statistically combining significantly heterogeneous data may be problematic. However, we were able to account for most of the heterogeneity in the secondary outcomes of interest by performing a sensitivity analysis and excluding specific trials from the secondary analysis. Exclusion of these trials did not meaningfully affect the pooled effect estimates. Further, our estimates of harm were derived from randomized studies of treatment efficacy. The decision to use only randomized trial data was made a priori and was based on the fact that rigorously conducted trials provide the most robust efficacy data. Consequently, we did not incorporate data from observational studies which may have enrolled more patients, provided longer follow-up, and been more reflective of harm associated with treatment. Lastly, the subgroup analysis and meta-regression we performed was underpowered, limited by the seven trials identified for this study. Therefore, the subgroup analysis is merely hypothesis-generating, and caution should be employed not to over-interpret the results.

## Conclusions

Our systematic review summarizes the current state of knowledge regarding aspirin effects on risk of MACE and bleeding. While less than definitive, the body of literature indicates, somewhat predictably, that aspirin does reduce the risk of cardiovascular events while causing a trend toward higher rates of bleeding and gastrointestinal complications. The trade-off of these benefits and harms, in absolute terms, suggests that the diabetes patient without cardiovascular disease lies somewhere between primary versus secondary prevention patients on the spectrum of benefit and risk. We now await the results of ACCEPT-D [39] and ASCEND [40] to help derive more definitive estimates of absolute benefit and harm associated with aspirin, and more refined patient-specific estimates of the risk-benefit trade-offs.

## Competing interests

The authors declare that they have no competing interests.

## Authors' contributions

First authorship is shared between SB and AAL as both had equal contribution to study concept and design, search of the literature, data extraction, data analyses, and the drafting and revision of the manuscript. DMR and WAG contributed to all aspects of the study, from conception and design, to analysis, and review of the final manuscript.

## Supplementary Material

Additional file 1 Detailed description of search strategy Click here for file
