# Supplementary material for: Aspirin effect on the incidence of major adverse cardiovascular events in patients with diabetes mellitus: a systematic review and meta-analysis
Source: Cardiovasc Diabetol. 2011 Apr 1;10:25. doi: 10.1186/1475-2840-10-25 (PMC3098148; doi:10.1186/1475-2840-10-25)
Supplement: Additional file 1 — Detailed description of search strategy [file 1475-2840-10-25-S1.DOC]

**Additional File 1.**

**Ovid MEDLINE(R)**

1. exp Primary Prevention/
2. primary prevention.ab,ti.
3. 1 or 2
4. exp Diabetes mellitus/
5. diabetes mellitus.ab,ti.
6. 4 or 5
7. exp Aspirin/
8. aspirin.ab,ti.
9. 7 or 8
10. 3 and 9
11. 6 and 9
12. 10 or 11
13. (controlled clinical trial or randomized controlled trial or meta analysis).pt.
14. (placebo* or random* or trial* or groups).ti,ab.
15. drug therapy.fs.
16. 13 or 14 or 15
17. limit 16 to animals
18. limit 16 to (animals and humans)
19. 17 not 18
20. 16 not 19
21. 12 and 20

**EMBASE (OVID)**

1. exp Primary Prevention/
2. primary prevention.ab,ti.
3. 1 or 2
4. exp Diabetes mellitus/
5. diabetes mellitus.ab,ti.
6. 4 or 5
7. exp Aspirin/
8. aspirin.ab,ti.
9. 7 or 8
10. 3 and 9
11. 6 and 9
12. 10 or 11
13. cross-over procedure/ or double-blind procedure/ or randomized controlled trial/ or single-blind procedure/
14. (allocat* or assign* or cross over* or crossover* or (double ADJ blind*) or factorial or placebo* or random* or (single ADJ blind*) or volunteer*).ti,ab.
15. 13 or 14
16. limit 15 to animals
17. limit 15 to (animals and humans)
18. 16 not 17
19. 15 not 18
20. 12 and 19

**The Cochrane Library – including Cochrane Database of Systematic Reviews (Cochrane Reviews), Database of Abstracts of Reviews of Effects (Other Reviews), Cochrane Central Register of Controlled Trials (Clinical Trials)**

1. **(aspirin):ti,ab,kw**
2. **(primary prevention):ti,ab,kw**
3. **(diabetes mellitus):ti,ab,kw**
4. **(#1 AND #2)**
5. **(#1 AND #3)**
6. **(#4 OR #5)**
7. Restricted to “Cochrane Reviews”, “Other Reviews”, and “Clinical Trials”

**BIOSIS**

1. “aspirin” in topic or title or major concepts
2. “primary prevention” in topic or title or major concepts
3. “diabetes mellitus” in topic or title or major concepts
4. #1 AND #2
5. #1 AND #3
6. #4 OR #5
7. Restricted to “All languages”, “Article”, “All literature types”, and “Humans”
